# The direction of restructuring of a Korea field epidemiology training program through questionnaire survey among communicable disease response staff in Korea

**DOI:** 10.4178/epih.e2017032

**Published:** 2017-07-24

**Authors:** Moo-Sik Lee, Kwan Lee, Ji-Hyuk Park, Jee-Young Hong, Min Young Jang, Byoung-Hak Jeon, Sang Yun Cho, Sun Ja Choi, Jeong Ik Hong

**Affiliations:** 1 Department of Preventive Medicine, Konyang University College of Medicine, Daejeon, Korea; 2 Department of Preventive Medicine, Dongkuk University College of Medicine, Gyeongju, Korea; 3 Division of Public Health Preparedness and Response, Korea Centers for Disease Control and Prevention, Cheongju, Korea

**Keywords:** Epidemiologists, Communicable diseases, Education, Surveys and questionnaires

## Abstract

We used a survey about the need for an educational training of infectious disease response staff in Korea Centers for Disease Control and Prevention (KCDC) and officer in metropolitan cities and provincial government to conduct field epidemiological investigation. The survey was conducted from January 25 to March 15, 2016. A total of 173 participants were selected from four different groups as follows: 27 clinical specialists, 22 Epidemic Intelligence Service (EIS) officers, 82 KCDC staff, and 42 local health department officials. Results revealed that 83% of KCDC staff and 95% of local health department officials agreed on the need for educational training to strengthen capability of personnel to conduct epidemic research and investigation. The level of their need for training was relatively high, while self-confidence levels of individuals to conduct epidemic research and investigation was low. It was concluded that there was a need to develop training programs to enhance the ability of public health officials, EIS officers, KCDC staff, and local health department personnel to conduct epidemic research and investigation.

## INTRODUCTION

Since the 2000s, the number of newly emerging and re-emerging infectious diseases has increased rapidly along with climate change. At the same time, the risk of new infections has increased because of greater probability of overseas infections being introduced to Korea as the number of foreign travelers increases owing to diversification of overseas travel opportunities and international trade [1].

Moreover, it is difficult to understand the domestic outbreak characteristics of emerging infectious diseases or those introduced from overseas, such as Middle East Respiratory Syndrome (MERS) and Ebola, and, although there is a dire need for specialists with accumulation of experience in dealing with various infectious diseases, current selection methods for Epidemic Intelligence Service (EIS) officers show little ability to discriminate and select highly competent resources for the job at hand. Moreover, because it is impossible to function as an EIS officer after being appointed and trained as an EIS officer, there are problems with no accumulation of human resources and expansion of organizational capacity.

In particular, Korea experienced a national health crisis in 2015 due to a MERS epidemic after introduction of MERS patients infected from the Middle East into Korea, and this national health crisis became a starting point in recognizing the great importance of establishing an epidemic defense system for preventing infectious diseases. Subsequent to the MERS epidemic, amendments to the regulations regarding prevention and management of infectious diseases established the legal basis for assigning 30 government EIS officers in the Ministry of Health and Welfare and 36 officers in local municipal, including city and provincial, government agencies, and, as such, a fundamental transformation is needed from the perspective of selecting and nurturing human resources to strengthen the epidemiological investigation competency of the Korea Centers for Disease Control and Prevention (KCDC).

EIS officers play a central role in the Korean epidemic defense system against various infectious diseases, and, from its initial pilot program in 1999 to 2015, most EIS officers have comprised public health physicians who have completed the basic training for becoming an EIS officer to serve their military duty as a medical officer. However, because of the nature of public health physicians, it was difficult to select physicians who specialized in infectious diseases, and EIS officers could function in that capacity for a limited time of only 2 years or so after training and assignment. After working in that capacity, most public health physicians who served as EIS officers went to work in their own field of specialty and the private sector, unrelated to public health, as soon as they were relieved of their duties as EIS officers. These physicians were assigned to EIS officer position for their 3 years mandatory military service.

Therefore, the existing EIS officer system showed organizational issues such as failure in both accumulation of human resources with experience and strengthening of the competency to respond against infectious diseases based on such experience.

A lack of EIS officers with specialized expertise was pointed out as one of the causes of the 2015 MERS epidemic, and, as a result, a decision was made to add a significant number of EIS officers as an important part of improving the national epidemic defense system. Consequently, the legal basis was established for assigning 30 government EIS officers in the KCDC and 36 local health department officials [2]. With the change of the existing system of EIS officers from being based on public health physicians to comprising professional government employees, the need for fundamental transformation of EIS officer training to strengthen the epidemiological investigation competency has been heightened from the perspective of selecting and nurturing human resources. In keeping with the change in those who will participate as EIS officers, a revision of the direction of education training courses for EIS officers is urgently required [3].

Accordingly, there is a need to establish base data on systematic improvement in nurturing epidemiological investigation specialists among infectious disease response staff at central (KCDC) and local municipal government levels. At the same time, there is also a need for curriculum reform and content development to strengthen the epidemiological investigation competency in KCDC staff and to address the change in those who will participate as EIS officers. Accordingly, the present study aimed to conduct a questionnaire survey on these topics, analyze the results, and introduce a summary of such findings. Moreover, the study also aimed to examine detailed requirements for improvement and strengthening of comprehensive education on epidemiological investigation, including the curriculum, methods, and contents.

## MATERIALS AND METHODS

To establish measures for nurturing the epidemiological investigation specialists, the present study selected 175 participants from 4 sectors consisting of 27 academic and clinical specialists, 22 EIS officers, 84 KCDC employees, and 42 local municipal health department officials. A questionnaire survey was conducted on the participants regarding the need for educational training on epidemiological investigation, opinions on educational operation when planning specialist nurturing, and the need for curriculum and competency for performing epidemiological investigation in the epidemiological investigation education program. A total of 173 participants responded to the questionnaire survey for a response rate of 98.9%. The questionnaire survey was conducted between January 25 and March 18, 2016. Analysis was performed using simple frequency and comparison of mean values.

## RESULTS

The total number of respondents was 173, consisting of 27 specialists (16%), 22 EIS officers (13%), 82 KCDC employees (47%), and 42 local municipal health department officials. There were more females (n= 96, 56%) than males (n= 77, 44%) among the respondents, while < 39 years old was the most common age group (n= 99, 57%) followed by 40-49 years (n= 46, 27%) and ≥ 50 years (n= 28, 16%). With respect to education level, bachelor’s degree was the most common response (n= 59, 34%) followed by master’s (n= 57, 33%) and doctorate (n= 57, 33%) degrees. With respect to affiliation, the responses appeared in the order of KCDC (n= 89, 51%), metropolitan city/provincial government (n= 55, 32%), university (n= 23, 13%), hospital (n= 4, 2%), and Incheon International Airport Quarantine Station (n= 2, 1%). Work period of less than 9 years (n= 105, 61%) was the most common response followed by 20-29 years (n= 30, 17%), 10-19 years (n= 30, 17%), and ≥ 30 years (n= 8, 5%), while type of work appeared in the order of healthcare (n= 60, 66%), nursing (n= 13, 14%), and administrative (n= 12, 13%) (Table 1).

**Figure 1.**
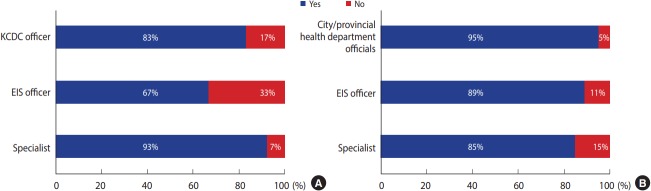
The needs of epidemiological training (A) for Korea Centers for Disease Control and Prevention (KCDC) staff and (B) officer of metropolitan cities and provincial governments. EIS, Epidemic Intelligence Service.

In the survey on the need for specialized education training on epidemiological investigation for KCDC employees and local municipal health department officials, 83% of all respondents (93% of specialists, 83% of KCDC employees, and 67% of EIS officers) responded “yes,” indicating a need for such training for KCDC employees, while 91% of all respondents (95% of local municipal health department officials, 89% of EIS officers, and 85% of specialists) responded “yes” in case of local municipal health department officials (Figure 1).

In the survey on educational operation when planning epidemiological investigation specialist nurturing, different responses were given depending on the target of educational training, as shown in Table 2. For educational training targeting KCDC employees, the mean number of hours per training session was 53.4 while the mean number of annual training sessions was 6.4. For interval between training sessions, 3 months was the most common response (n= 52, 41%) while the combination of lectures and practical exercises (n= 121, 95%) was the most frequent response given as the most appropriate training method. The most common responses to the number of participants per training session and the department in charge of training were 10-19 participants (n= 83, 65%) and self-operation by KCDC (n= 57, 45%), respectively. With respect to the need for training before assigning tasks to new employees, 97% (n=124) responded “yes” with 2 weeks being the most common response for the length of training prior to assigning tasks to new employees (n= 49, 40%).

For educational training targeting local municipal health department officials, the mean number of hours per training session was 44.1 while the mean number of annual training sessions was 6.8. For interval between training sessions, 3 months was the most common response (n= 46, 53%) while the combination of lectures and practical exercises (n=75, 86%) was the most frequent response given as the most appropriate training method. The most common responses to the number of participants per training session and the department in charge of training were 10-19 participants (n= 41, 47%) and self-operation by KCDC (n= 48, 55%), respectively. With respect to the need for training before assigning tasks to new employees, 93% (n=82) responded “yes” with within 1 week being the most common response for the length of training prior to assigning tasks to new employees (n= 32, 39%).

The results from the survey on assessment of the impact of education training on epidemiological investigation competency and the required training curriculum, graded on a maximum of 5.0 points, are shown in Table 3. In the survey on assessment of the impact of education training on epidemiological investigation competency conducted among KCDC employees, the highest scores were found in the items “water-borne and food-borne infectious diseases,” “infectious diseases targeted for vaccination,” and “chronic infectious diseases” with 3.0 points each. Within the category of infectious disease epidemiology, the highest scores were found in “understanding the concept and processes of epidemiological studies” and “use of personal protective equipment” with 3.0 points each. In the category of data collection and analysis, the highest score of 3.1 points was found in “understanding the scale of data and inputting data.” For sample collection and laboratory testing, “collection, packaging, and transportation of samples” and “understanding and interpreting the test results” showed the highest scores of 2.9 points each while, for other capabilities, “creating documents” had the highest score of all with 3.5 points. In the survey on the need for training program conducted on KCDC employees, “emerging infectious disease” had the highest score of 4.4 points in the category of understanding of infectious diseases while “understanding the concept and processes of epidemiological studies” had the highest score of 4.5 points in the category of infectious disease epidemiology. In the category of data collection and analysis, “descriptive analysis of epidemiological studies” had the highest score of 4.1 points while, in the category of sample collection and laboratory testing, “collection, packaging, and transportation of samples” and “understanding and interpreting the test results” showed the same scores of 3.8 points. Meanwhile, among other capabilities, the highest score of 4.0 points was found in “presentation of epidemiological studies data and results to decision-makers and stakeholders.”

In the survey on assessment of the impact of education training on epidemiological investigation competency conducted for local municipal health department officials, “infectious diseases targeted for vaccination” had the highest score of 2.8 points in the category of understanding of infectious diseases while “use of personal protective equipment” had the highest score of 2.8 points in the category of infectious disease epidemiology. For data collection and analysis, “understanding the scale of data and inputting data” showed the highest score of 2.6 points while, for sample collection and laboratory testing, “collection, packaging, and transportation of samples” and “understanding and interpreting the test results” showed the same scores of 2.5 points. Meanwhile, for other capabilities, “creating documents” had the highest score of all with 3.2 points. In the survey on the need for training program conducted among local municipal health department officials, “water-borne and food-borne infectious diseases” and “emerging infectious diseases” had the highest scores in the category of understanding of infectious diseases while “understanding the concept and processes of epidemiological studies” had the highest score in the category of infectious disease epidemiology, all with scores of 4.2 points. In the category of data collection and analysis, “descriptive analysis of epidemiological studies” had the highest score of 4.0 points while, in the category of sample collection and laboratory testing, “collection, packaging, and transportation of samples” and “understanding and interpreting the test results” showed the same high score of 3.8 points each. For other capabilities, “presentation of epidemiological studies data and results to decision-makers and stakeholders” showed the highest score of 4.0 points (Table 3).

## DISCUSSION

KCDC began operating Korea Field Epidemiology Program (K-FETP) as a pilot program in 1999. Since that time, it has trained public health physicians and government workers to produce 15-20 EIS officers per year. Public health physicians who completed the 4-week basic EIS officer training, within the curriculum, have been appointed as EIS officers and assigned to central and local health department posts as EIS officers [4,5].

Meanwhile, the Field Management Training Program, supervised and operated by Korea Human Resource Development Institute for Health and Welfare, is also conducting epidemiological investigation training for infectious disease response staff in various levels of local government, but specific time has not been allocated exclusively for strengthening epidemiological investigation competency [6,7].

This is counterevidence to the point that K-FETP operated by KCDC, as the only program in Korea for developing EIS officers, is a very important training program for enhancing the ability to respond to infectious diseases on a national level [8]. Moreover, the fact that the significantly increased number of EIS officers after the MERS epidemic included professional government workers and not existing public health physicians also heightened the need for EIS officer educational training reform. Survey results on need for educational training to strengthen epidemiological investigation competency with 83% of KCDC employees and 95% of local health department officials demonstrate a high demand for reform by those who participate in such educational training. It is determined that these results reflect the fact that KCDC and health department workers who were assigned to the 2015 MERS epidemic recognized on their own a need to grow the ability to perform epidemiological investigation, which was a vital process in responding to infectious diseases. Educational training for strengthening epidemiological investigation competency, targeting KCDC and local health department workers, has the goal of cultivating practical epidemiological investigation competency in local health department officials and healthcare professionals in charge of epidemiological investigation, except EIS officers. Because educational training for strengthening epidemiological investigation competency still lacks the details for its purpose, participants in such training, core competencies, educational goals, educational topics, methods, and materials, it would be necessary to reference K-FETP when considering expected problems and operational issues that may arise when such training program is implemented. It is also anticipated that the survey results from the present study on various categories about educational training on epidemiological investigation will serve as important data in selecting the areas of focus in the process of future educational training program reform.

## CONCLUSION

In conclusion, the need for epidemiological investigation educational training reform and levels of satisfaction in various subcategories of the curriculum found in the present study can be helpful in developing a new educational training program for epidemiological investigation to be implemented in the future, and it is believed that such findings will also make a significant contribution in strengthening the competency of EIS officers who will form the basis of a national epidemic-defense system.

## Figures and Tables

**Table 1.** General characteristics of surveyed participants (n=173)

Category	n (%)	Category	n (%)
Respondent		Affiliation	
Specialist	27 (16)	University	23 (13)
EIS officer	22 (13)	Hospital	4 (2)
KCDC staff	82 (47)	KCDC	89 (52)
Local officials	42 (24)	Quarantine station	2 (1)
Sex		Local government	55 (32)
Male	77 (44)	Work period (yr)	
Female	96 (56)	≤9	105 (61)
Age (yr)		10-19	30 (17)
≤39	99 (57)	20-29	30 (17)
40-49	46 (27)	≥30	8 (5)
≥50	28 (16)	Series of class	
Education		Administration	12 (13)
Bachelor's degree	59 (34)	Healthcare	60 (66)
Master's degree	57 (33)	Nursing	13 (14)
Doctor's degree	57 (33)	Others	6 (7)

EIS, Epidemic Intelligence Service; KCDC, Korea Centers for Disease Control and Prevention.

**Table 2.** The opinions about the operational of educational training program

Category	A target of educational training		
	KCDC staffs	Local officials	
Time per educational training (hr)		
Mean士SD	53.4±51.6	44.1±45.5
Min-Max	1-240	2-240
Median	40	30
No. of annual educational training (times)		
Mean士SD	6.4±21.3	6.8±21.3
Min-Max	1-240	1-200
Median	4	4
Interval among educational training		
1 mo	12 (10)	6 (7)
3 mo	52 (41)	46 (53)
6 mo	44 (35)	23 (26)
1 yr	18 (14)	12 (14)
The most appropriate educational training methods		
Lectures and exercises in parallel	121 (95)	75 (86)
Virtual training	7 (5)	11 (13)
Lectures or online training	0 (0)	1 (1)
No. of people who participate per educational training		
≤9	21 (16)	14 (16)
10-19	83 (65)	41 (47)
20-29	22 (17)	28 (32)
≥30	2 (2)	4 (4)
Others	-	1 (1)
Managing department		
Self-operation of KCDC	57 (44)	48 (55)
Contracting out of educational training	35 (28)	28 (32)
Korea Human Resource development institute for Health & Welfare	35 (28)	11 (13)
Need of educational training before arranging task in new employee		
Yes	124 (97)	82 (93)
No	4 (3)	6 (7)
Total time of educational training before arranging task in new employee (wk)		
≤1	34 (28)	32 (39)
2	49 (40)	26 (32)
3	14 (11)	13 (16)
4	26 (21)	11 (13)

Values are presented as number (%).

Min, minimum; Max, maximum; SD, standard deviation; KCDC, Korea Centers for Disease Control and Prevention.

**Table 3.** Impact of educational training on epidemiological investigation and the need of programs in educational training

Category	Impact of education on epidemiological investigation		Need of programs	
	KCDC staffs	Local officials	KCDC staffs	Local officials
Understanding of infectious diseases				
Water-borne infectious disease	3.0±0.9	2.7±0.9	4.2±0.7	4.2±0.7
Infectious disease targeted for the vaccination	3.0±1.0	2.8±1.0	4.2±0.8	4.1±0.7
Chronic infectious disease (HIV, tuberculosis, etc.)	3.0±0.9	2.7±0.9	4.0±0.8	4.0±0.8
Zoonosis (brucellosis etc.)	2.7±1.0	2.4±1.0	4.0±0.8	3.9±0.8
Vector-borne infection disease (malaria, tsutsugamushi, etc.)	2.9±1.0	2.6±0.9	4.2±0.7	4.0±0.7
Emerging infectious disease (AI, SARS, Ebola, etc.)	2.7±1.0	2.6±1.1	4.4±0.7	4.2±0.8
Infectious disease epidemiology				
The concept of epidemiological studies and process understanding	3.0±1.0	2.6±1.0	4.5±0.7	4.2±0.8
Use of personal protective equipment	3.0±1.2	2.8±1.1	4.4±0.9	4.1±0.7
Interview participants	2.7±1.0	2.6±0.9	4.3±0.9	4.1±0.8
Data collection and analysis				
To understand scale of data and to input data	3.1±1.1	2.6±1.1	3.9±1.0	3.9±0.9
A descriptive analysis of epidemiological studies	3.0±1.0	2.5±1.0	4.1±0.9	4.0±0.9
Statistical analysis	2.8±1.0	2.3±1.1	3.9±1.1	3.8±1.0
Sample collection and laboratory testing				
Collecting of the samples, packaging, transportation	2.9±1.0	2.5±0.9	3.8±1.0	3.8±1.0
To understand the results of the scan of a specimen and analysis	2.9±1.1	2.5±1.0	3.8±1.0	3.8±0.9
Other capabilities				
Creating documents (table, chart, etc.)	3.5±1.0	3.2±1.0	3.5±1.1	3.7±1.0
Presentation of the result of survey	3.0±0.9	2.7±1.0	4.0±1.0	4.0±0.8
Writing the massage on issues of public interest	2.7±0.9	2.7±0.9	3.8±1.1	3.9±0.9
Writing press releases and media response	2.8±0.9	2.8±0.9	3.9±1.1	3.9±0.9

Values are presented as mean±standard deviation.

KCDC, Korea Centers for Disease Control and Prevention; HIV, human immunodeficiency virus; SARS, severe acute respiratory syndrome.

## References

[1] Korea Centers for Disease Control and Prevention (2016). Infectious disease surveillance yearbook, 2015.

[2] National Law Information Center (2016). Infectious Disease Control and Prevention Act: Article 60-2. http://www.law.go.kr/main.html.

[3] Oh SH (2010). Men political science.

[4] Kwon GY, Moon S, Kwak W, Gwack J, Chu C, Youn SK (2013). Epidemic intelligence service officers and field epidemiology training program in Korea. Osong Public Health Res Perspect.

[5] Jun B (2015). Middle East respiratory syndrome outbreak and infectious disease control in Korea. J Korean Med Assoc.

[6] Korea Centers for Disease Control and Prevention (2016). Infectious diseases control program guidelines.

[7] Park NR, Jeong IS, Lee JG, Kim YT, Chun JH, Kim KS (2004). Evaluation of field epidemiology specialist training program based on the satisfaction and the changes of educational needs. Korean J Prev Med.

[8] Dongkuk University (2011). Development of educational course and training manual for field epidemiologist training program during 2011-2015.

