# Supplementary material for: The direction of restructuring of a Korea field epidemiology training program through questionnaire survey among communicable disease response staff in Korea
Source: Epidemiol Health. 2017 Jul 24;39:e2017032. doi: 10.4178/epih.e2017032 (PMC5675987; doi:10.4178/epih.e2017032)
Supplement: Supplementary file 1 [file epih-39-e2017032-supplementary.pdf]

Volume: 39, Article ID: e2017032s

https://doi.org/10.4178/epih.e2017032

감염병 대응인력 대상 설문조사를 통한 역학조사교육의 개편방향

이무식, 이관[*], 박지혁[*], 홍지영, 장민영, 전병학[**], 조상현[**], 최선자[**], 홍정익[**]

건양대학교 의과대학 예방의학교실, 동국대학교 의과대학 예방의학교실[*]

질병관리본부 위기대응총괄과[**]

The Direction of Restructuring of Korea Field Epidemiology Training Program through Questionnaire Survey among Communicable Disease Response Staff in Korea

Moo-Sik Lee, Kwan Lee[*], Jee-Hyuk Park[*], Jee-Young Hong, Min-Young Jang, Byoung-Hak Jeon[**], Sang-Yun Cho[**], Sun-Ja Choi[**], JeongIk Hong[**]

Department of Preventive Medicine, College of Medicine, Konyang University

Department of Preventive Medicine, College of Medicine, Dongkuk University[*]

Division of Public Health Preparedness and Response, Centers for Disease Control and Prevention, Republic of Korea[**]

Running Title : **Restructuring of a Korea Field Epidemiology Training Program**

Corresponding author : Moo-Sik Lee

Department of Preventive Medicine, College of Medicine, Konyang University

158, Gwanjeodong-ro, Seo-gu, Daejeon 35365, Korea

주소: (35365) 대전광역시 서구 관저동로 158 건양대학교 의과대학 예방의학교실

Tel: +82-42-600-6404, Fax: +82-42-600-6401, E-mail: mslee@konyang.ac.kr

Volume: 39, Article ID: e2017032s

https://doi.org/10.4178/epih.e2017032

Abstract

Objectives: We used a survey about the need for an educational training of infectious disease response staff in Korea Centers for Disease Control and Prevention (KCDC) and officer in metropolitan cities and provincial government to conduct field epidemiological investigation.

Methods: The survey was conducted from January 25 to March 15, 2016. A total of 173 participants were selected from four different groups as follows: 27 clinical specialists, 22 Epidemic Intelligence Service (EIS) officers, 82 KCDC staff, and 42 local health department officials.

Results: Results revealed that 83% of KCDC staff and 95% of local health department officials agreed on the need for educational training to strengthen capability of personnel to conduct epidemic research and investigation. The level of their need for training was relatively high, while self-confidence levels of individuals to conduct epidemic research and investigation was low.

Conclusions: It was concluded that there was a need to develop training programs to enhance the ability of public health officials, EIS officers, KCDC staff, and local health department personnel to conduct epidemic research and investigation.

Keywords: field epidemiological investigation, infectious disease, educational training

서 론

2000년대에 접어들면서 기후 변화와 더불어 신종 및 재출현 감염병이 급증하고 있으며, 국가간 교역과 해외여행의 기회 다양화 등으로 해외여행객이 늘어남에 따라 해외 발생 감염병의 국내 유입 가능성이 증대 새로운 감염위협이 증가되고 있다[1].

또한 메르스, 에볼라 등 해외 유입 및 신종 감염병의 경우 국내 발생 특성 등을 파악하는데 어려움이 있으며, 여러 감염병에 대한 경험이 축척된 전문가의 필요성이 절실하나, 현재의 역학조사관 선방 방안으로는 유능한 직종의 자원을 선발하는데 변별력이 없으며, 역학조사관 임명 및 교육 이후에는 지속적인 역학조사관으로서의 활동이 불가하여, 인적자원의 축적 및 조직의 역량으로 확산되지 못하는 문제점이 있다.

특히, 2015년 중동 지역에서 감염된 메르스 환자의 국내 유입 후 유행으로 국가적인 보건위기 상황을 겪었으며, 이 과정에서 감염병을 적극적으로 막기 위한 방역 시스템의 마련이 매우 중요

Volume: 39, Article ID: e2017032s

https://doi.org/10.4178/epih.e2017032

함을 확인하는 계기가 되었다. 메르스 유행이루 감염병의 예방 및 관리에 관한 법률 개정으로 보건복지부에 30명, 시도에 36명 이상의 공무원 역학조사관 배치의 법적 근거가 마련됨에 따라 질병관리본부의 역학조사 역량강화를 위한 인력 선방 및 양성 측면에서 근본적인 변혁이 필요하다.

다양한 감염병에 대한 국내 방역체계의 중심에는 역학조사관이 있으며, 1999년 시범사업 이후 2015년까지 역학조사관의 대부분은 역학조사관 기본교육을 수료한 공중보건의로 구성되었다. 그러나 공중보건의의 특성상 감염병에 대한 전문성을 갖춘 전문적 의사 자원의 선발이 힘들며, 역학조사관 교육과 임명 이후 2년여의 한시적인 역학조사관 활동을 수행할 수밖에 없었다. 활동 이후 대부분의 공중보건의 역학조사관은 전역과 동시에 공중보건과 무관한 자신의 전공분야 및 민간의 영역에 근무하였다. 따라서 기존의 역학조사관 제도는 경험을 갖춘 인적자원의 축적과 이를 통한 감염병 대응 역량의 강화로 이어지지 못하는 구조적인 문제점이 있었다.

전문성을 갖춘 역학조사관의 부족이 2015년 메르스 사태의 주요한 원인 중 하나로 제시되었고, 국가방역체계를 개선하기 위한 중요한 부분으로서 역학조사관의 대폭적인 충원 결정이 있었다. 이 결과로 보건복지부(질병관리본부)에 30명, 시•도에 34명 이상의 공무원 역학조사관 배치에 대한 법적근거가 마련되었다[2]. 기존 공중보건의 중심의 역학조사관 제도가 전문직 공무원으로 구성된 역학조사관으로 바뀌게 됨에 따라, 역학조사 역량강화를 위한 인력 선발 및 양성 측면에서 역학조사관 교육에 대한 근본적인 변혁 필요성이 높아졌다. 역학조사관 참여대상 변화에 따라 역학조사관 교육훈련과정에 대한 개편방향을 시급히 확정할 필요가 대두되었다[3].

이에 역학조사와 관련된 중앙(질병관리본부) 및 시·도 감염병 대응인력을 대상으로 역학조사 전문인력 양성을 위한 제도개선의 근거자료를 마련하는 동시에 역학조사관 참여대상 변화 및 질병관리본부 등 직원의 역학조사 역량강화를 위한 교육과정 개편 및 컨텐츠 개발이 필요한데 이에 대한 설문조사를 통하여 그 결과를 분석, 요약하여 본고에 소개하고자 한다. 또한 교육과정, 방법, 내용 등 포괄적인 역학조사 교육 강화 및 개선이 필요한 세부 내용에 대해 살펴보고자 한다.

대상 및 방법

역학조사 전문인력 육성방안을 수립하기 위해 학계 및 임상 전문가 27명, 역학조사관 22명, 질병관리본부 직원 84명, 시·도 공무원 42명 등 4가지 분야별 대상자 총 175명를 선정하여, 역학조사 교육훈련의 필요성, 전문인력 육성 계획 시 교육 운영에 대한 의견, 역학조사 교육 프로그램의

Volume: 39, Article ID: e2017032s

https://doi.org/10.4178/epih.e2017032

역학조사 수행역량 및 교육프로그램의 교과목 필요성 등에 대해 설문조사를 수행하였다. 설문조사 응답자는 총 173명으로 응답률은 98.9%였다. 미응답자는 질병관리본부 직원중 3명이었다. 설문조사는 2016년 1월 25일-3월 18일까지 수행하였다. 분석은 단순 빈도 및 평균치 비교 등으로 이루어졌다.

결 과

전체 응답자수는 173명으로 전문가 27명(15%), 역학조사관 22명(13%), 질병관리본부 직원 81명(46%), 시·도 공무원 42명(24%)이 참여하였다. 여자 95명(55%), 남자 77명(45%)로 여성이 더 많았으며, 39세 이하가 99명(58%)으로 가장 많고, 40-49세 45명(26%), 50세 이상이 27명(15%) 순이었다. 학력분포는 학사 59명(34%), 석사 57명(33%), 박사 57명(33%)이었으며, 질병관리본부 소속 88명(51%), 광역 시·도 소속 55명(32%), 대학 소속 23명(13%), 병원 소속 4명(2%), 인천공항검역소 소속 2명(1%) 순 이었다. 9년 이하 근무자 101명(61%)로 가장 높았고 20-29년이 30명(18%), 10-19년이 27명(16%), 30년 이상이 8명(5%) 순으로 나타났으며, 보건직렬 60명(66%)으로 가장 많았고, 간호직렬 13명(14%), 행정직렬 12명(13%) 순 이었다(Table 1).

Table 1. General characteristics of surveyed subjects (N=173)

Category	N (%)	Category	N (%)
Respondent		Affiliation	
Specialist	27 (15)	University	23 (13)
EIS officer*	22 (13)	Hospital	4 (2)
KCDC staffs	82 (46)	KCDC†	88 (51)
Local officials	42 (24)	Quarantine Station	2 (1)
Gender		Local government	55 (32)
Male	77 (45)	Work period (years)	
Female	95 (55)	≤ 9	101 (61)
Age (years)		10-19	27 (16)
≤ 39	99 (58)	20-29	30 (18)

Volume: 39, Article ID: e2017032s

https://doi.org/10.4178/epih.e2017032

40-49	45 (26)	≥ 30	8 (5)
≥ 50	27 (16)	Series of class	
Education		Administration	12 (13)
Bachelor's degree	59 (34)	Health care	60 (66)
Master´s degree	57 (33)	Nursing	13 (14)
Doctor's degree	57 (33)	Others	6 (7)

[*] EIS officer : Epidemic Intelligence Service officer
[†] KCDC : Korea Centers for Disease Control and Prevention

질병관리본부 직원 및 시·도 공무원을 대상으로 하는 역학조사 전문인력 교육훈련의 필요성에 대한 의견을 조사한 결과, 질병관리본부 직원에 대한 역학조사 교육훈련의 필요성에 대해 전체중 83% (전문가 93%, 질병관리본부 근무 직원 83%, 역학조사관 67%)가 필요하다고 응답하였고, 시·도 공무원의 경우 전체 중 91% (시·도 공무원 95%, 역학조사관 90%, 전문가 85%)가 역학조사 교육훈련이 필요하다고 응답하였다(Figure 1).

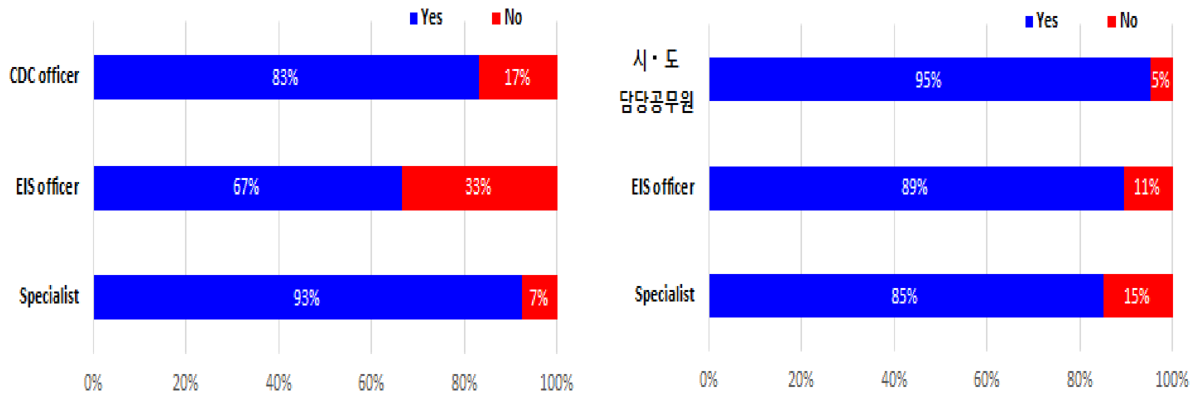

(a) (b)

Figure 1. The needs of epidemiological training (a) for KCDC Staff and (b) officer of metropolitan cities and provincial governments

역학조사 전문인력 육성 계획 시 교육 운영에 대한 의견을 조사 결과 교육대상에 따라 (표2)와 같은 응답을 얻을 수 있었다. 질병관리본부 직원을 대상으로 하는 교육일 경우, 1회 교육 시수는 평균 53.4시수였고 연간 교육 시행 횟수는 평균 6.4회로 의견이 도출되었다. 교육종료 후 다음 교육까지의 기간은 3개월이 52명(41%)으로 가장 많았고 가장 적합한 교육훈련 방법은 강의와 실습병행이 121명(95%)으로 가장 높았다. 회당 교육훈련 인원수는 11-20명이 83명(65%)으로 가

장 높았고 교육훈련 주관기관은 질병관리본부 자체 시행이 57명(45%)으로 높았다. 신입직원 업무 배치 전 교육훈련 필요성은 응답자 124명(97%) 필요하다고 응답하였고 신입직원 업무 배치 전 총 교육기간은 2주가 49명(40%)으로 가장 높았다.

시・도 공무원을 대상으로 하는 교육일 경우, 1회 교육 시수는 평균 44.1시수였고 연간 교육 시행 횟수는 평균 6.8회로 조사되었다. 교육종료 후 다음 교육까지의 기간은 3개월이 46명(53%)으로 가장 많았고 가장 적합한 교육훈련 방법은 강의와 실습병행이 75명(86%)으로 가장 높았다. 회당 교육훈련 인원수는 11-20명이 41명(47%)으로 가장 높았고 교육훈련 주관기관은 질병관리본부 자체시행이 48명(55%)으로 높았다. 신입직원 업무 배치 전 교육훈련 필요성은 응답자 82명(93%)이 필요하다고 응답하였고 신입직원 업무 배치 전 총 교육기간은 1주 이내가 32명(39%)으로 가장 높았다.

Table 2. The Opinions about the operational of educational training program

Category	A target of educational training	
	KCDC staffs	Local officials
Time per educational training		
mean±sd	53.4±51.6	44.1±45.5
min-max	1-240	2-240
median	40	30
Number of annual educational training (times)		
mean±sd	6.4±21.3	6.8±21.3
min-max	1-240	1-200
median	4	4
Interval among educational training (n, %)		
1 months	12(10)	6(7)
3 months	52(41)	46(53)
6 months	44(35)	23(26)
1 year	18(14)	12(14)
The most appropriate educational training methods (n, %)		
Lectures and exercises in parallel	121(95)	75(86)
Virtual training	7(6)	11(13)
Lectures or online training	0(0)	1(1)

Number of people who participate per educational training (n, %)		
≤ 10	21(16)	14(16)
11-20	83(65)	41(47)
21-30	22(17)	28(32)
≥ 31	2(2)	4(5)
Others	--	1(1)
Managing department (n, %)		
Self-operation of KCDC	57(45)	48(55)
Contracting-out of educational training.	35(28)	28(32)
Korea Human Resource development institute for Health & Welfare	35(28)	11(13)
Need of educational training before arranging task in new employee (n, %)		
Yes	124(97)	82(93)
No	4(3)	6(7)
Total time of educational training before arranging task in new employee (n, %)		
≤ 1 week	34(28)	32(39)
2 weeks	49(40)	26(32)
3 weeks	14(11)	13(16)
4 weeks	26(21)	11(13)

역학조사 교육 프로그램의 역학조사 수행역량에 미치는 영향력에 대한 평가와 필요한 교과목에 대하여 조사한 결과는(5점 만점) 표3과 같은 응답을 얻을 수 있었다(Table 3). 질병관리본부 직원을 대상으로 한 역학조사 교육 프로그램의 역학조사 수행역량에 대한 영향을 평가한 결과, 감염병에 대한 기본적 이해 중 “수인성·식품매개질환 감염병”, “예방접종 대상 감염병”과 “만성감염병”에 대한 평가가 3.0점으로 가장 높았다. 감염병 유행 역학조사 중 “역학조사의 개념과 과정을 이해하기”와 개인보호장비 사용법”에 대한 평가가 3.0점으로 가장 높았고, 자료 수집 및 분석하기에서는 “조사 자료의 척도 이해와 자료 입력를 이해하고 자료 입력하기”가 3.1점으로 높게 나타났다. 검체 채집 및 실험실 검사에서는 “검체의 채집, 수송용기 포장, 수송하기”와 “검체의 검사 결과를 이해하고 해석하기”가 2.9점으로 높게 나타났으며, 기타 역량 중 “문서 작성하기”가 전 부분에서 가장 높은 3.5점으로 평가되었다. 질병관리본부 직원을 대상으로 한 역학조사 교육 프로그램의 교과목 필요성에 대한 조사 결과, 감염병에 대한 기본적 이해 중 “신종감염병”에 대한 필요성이 4.4점으로 가장 높게 응답하였고, 감염병 유행 역학조사 중 “역학조사의 개념과 과정을 이해하기”가 4.5점으로 가장 높았다. 자료 수집 및 분석하기에서는 “기술적 역학연구 수행하기”가 4.1점으로 높게 나타났고, 검체 채집 및 실험실 검사에서는 “검체의 채집, 수송용기 포장, 수송하기”와 “검체의 검사 결과를 이해하고 해석하기”가 3.8점으로 동일하게 나타났으며, 기타

Volume: 39, Article ID: e2017032s

https://doi.org/10.4178/epih.e2017032

역량 중 “의사결정자와 이해관계자에게 감염병 유행 역학조사의 자료와 결과를 발표하기”가 4.0점으로 높은 필요도를 보였다.

시ㆍ도 공무원을 대상으로 한 역학조사 교육 프로그램의 역학조사 수행역량에 대한 평가에 대한 전체 조사 결과, 감염병에 대한 기본적 이해 중 “예방접종 대상 감염병”에 대한 평가가 2.8점으로 가장 높았고, 감염병 유행 역학조사 중 “개인보호장비 사용법”에 대한 평가가 2.8점으로 가장 높았다. 자료 수집 및 분석하기에서는 “조사 자료의 척도를 이해하고 자료 입력하기”가 2.6점으로 높게 나타났고, 검체 채집 및 실험실 검사에서는 “검체의 채집, 수송용기 포장, 수송하기”와 “검체의 검사 결과를 이해하고 해석하기”가 2.5점으로 높게 나타났으며, 기타 역량 중 “문서 작성하기”가 전 부분에서 가장 높은 3.2점으로 평가되었다. 시ㆍ도 공무원을 대상으로 한 역학조사 교육 프로그램의 교과목 필요성에 대한 전체 조사 결과, 감염병에 대한 기본적 이해 중 “수인성·식품매개질환 감염병”과 “신종감염병”에 대한 필요성이 4.2점으로 가장 높게 응답하였고, 감염병 유행 역학조사 중 “역학조사의 개념과 과정을 이해하기”가 4.2점으로 가장 높았다. 자료 수집 및 분석하기에서는 “기술적 역학연구 수행하기”가 4.0점으로 높게 나타났고, 검체채집 및 실험실 검사에서는 “검체의 채집, 수송용기 포장, 수송하기”와 “검체의 검사 결과를 이해하고 해석하기”가 3.8점으로 동일하게 나타났으며, 기타 역량 중 “의사결정자와 이해관계자에게 감염병 유행 역학조사의 자료와 결과를 발표하기”가 4.0점으로 높은 필요도를 보였다(Table 3).

Table 3. Impact of educational training on epidemiological investigation and the need of programs in educational training

Category	Impact of education on epidemiological investigation		Need of programs	
	KCDC staffs	Local officials	KCDC staffs	Local officials
	mean ± sd	mean ± sd	mean ± sd	mean ± sd
I. Understanding of infectious diseases				
1. Water-bone infectious disease	3.0± 0.9	2.7± 0.9	4.2± 0.7	4.2± 0.7
2. Infectious disease targeted for the vaccination	3.0± 1.0	2.8± 1.0	4.2± 0.8	4.1± 0.7
3. Chronic infectious disease (HIV, tuberculosis etc.)	3.0± 0.9	2.7± 0.9	4.0± 0.8	4.0± 0.8
4. Zoonosis (brucellosis etc.)	2.7± 1.0	2.4± 1.0	4.0± 0.8	3.9± 0.8
5. Vector-borne infection disease	2.9± 1.0	2.6± 0.9	4.2± 0.7	4.0± 0.7

Volume: 39, Article ID: e2017032s

https://doi.org/10.4178/epih.e2017032

(malaria, tsutsugamushi etc.)				
6. Emerging infectious disease (AI, SARS, Ebola etc.)	2.7± 1.0	2.6± 1.1	4.4± 0.7	4.2± 0.8
II. Infectious disease epidemiology				
1.The concept of epidemiological studies and process understanding	3.0± 1.0	2.6± 1	4.5± 0.7	4.2± 0.8
2. Use of personal protective equipment	3.0± 1.2	2.8± 1.1	4.4± 0.9	4.1± 0.7
3. Interview subjects	2.7± 1.0	2.6± 0.9	4.3± 0.9	4.1± 0.8
III. Data collection and analysis				
1. To understand scale of data and to input data.	3.1± 1.1	2.6± 1.1	3.9± 1.0	3.9± 0.9
2. A descriptive analysis of epidemiological studies.	3.0± 1.0	2.5± 1.0	4.1± 0.9	4.0± 0.9
3. Statistical analysis	2.8± 1.0	2.3± 1.1	3.9± 1.1	3.8± 1.0
IV. Sample collection and laboratory testing				
1. Collecting of the samples, packaging, transportation	2.9± 1.0	2.5± 0.9	3.8± 1.0	3.8± 1.0
2. To understand the results of the scan of a specimen, and analysis	2.9± 1.1	2.5± 1.0	3.8± 1.0	3.8± 0.9
V. Other capabilities				
1. Creating documents (table, chart etc.)	3.5± 1.0	3.2± 1.0	3.5± 1.1	3.7± 1.0
2. Presentation of the result of survey	3.0± 0.9	2.7± 1.0	4.0± 1.0	4.0± 0.8
3.Writing the massage on issues of public interest	2.7± 0.9	2.7± 0.9	3.8± 1.1	3.9± 0.9
4. Writing Press Releases and Media response	2.8± 0.9	2.8± 0.9	3.9± 1.1	3.9± 0.9

고 찰

질병관리본부는 1999년 시범사업부터 시작된 역학조사관 교육과정(Korea Field Epidemiology Program; K-FETP)의 운영을 통해 공중보건의 및 공무원을 대상으로 매년 15-20여명의 역학조사관을 교육하여 왔으며, 교육과정 중 4주간의 역학조사관 기본교육을 이수한 공중보건의사를 역학조사관으로 임명하여 중앙부처와 시·도의 역학조사관 인력으로 배출하여 왔다[4,5].

한편 한국보건복지인력개발원에서 담당·운영하고 있는 감염병전문가 교육(Field Management Training Program)을 통해 시·도 및 시·군·구의 역학조사반원인 감염병 대응요원에 대한 감염병 대응역량을 교육하고 있으나, 역학조사 역량 강화를 위한 전담 교육시간은 별도로 배정되어 있지는 않았다[6, 7].

이는 질병관리본부가 운영하고 있는 역학조사관 교육과정(K-FETP)이 역학조사관을 양성하는

국내 유일한 교육 프로그램으로서 감염병 대응의 국가적 능력을 제고하는데 있어 매우 중요한 교육과정이라는 점을 반증한다[8]. 또한 메르스 사태 이후 대폭 충원된 역학조사관이 기존의 공중보건의가 아닌 전문직 공무원이라는 점은 이러한 역학조사관 교육과정의 개편 필요성을 크게 높이고 있다. 질병관리본부 및 시도 직원 대상 역학조사 역량강화 교육의 필요성을 조사한 결과 질병관리본부 직원들의 경우 83%, 시도 직원의 경우 95%가 교육이 필요하다고 응답하는 등 교육 수요자의 개편 요구가 높은 것을 알 수 있었다. 이러한 결과는 2015년 메르스 유행 사태에 투입된 질병관리본부, 시·도 직원들이 감염병 대응에 있어 핵심활동인 역학조사를 수행함에 있어 실무역량을 키울 필요가 있음을 본인 스스로 인지한 것으로 판단된다. 질병관리본부 및 시도 직원을 대상으로 한 역학조사 역량강화 교육과정은 역학조사관을 제외한 보건당국 전문인력 중 질병관리본부 역학조사 주관부서에 근무하는 직원과 시도의 역학조사 담당자들을 대상으로 역학조사 실무역량을 배양하는 것을 목적으로 한다. 질병관리본부 및 시도 직원대상 역학조사 역량강화 교육과정은 아직 교육과정의 목적, 대상, 핵심 역량, 교육목표, 교육주제, 교육방법, 교육자료 등에 대한 구체적인 모습을 갖추지 못했기 때문에 향후 이 과정이 시행될 경우 발생 가능한 예상 문제점과 운영상 고려할 점은 역학조사관 교육프로그램(K-FETP) 사례를 참고할 필요가 있을 것이다. 이 조사에서 수행된 역학조사관 교육과정에서 진행된 교과목에 대한 설문결과는 앞으로의 교육프로그램 개편과정에서 집중하여야 되는 부분의 선택에 있어서 중요한 자료로 사용될 것으로 예상된다.

결 론

결론적으로 이 조사연구를 통해 확인한 역학조사관 교육 개편의 필요성과 세부 교육과정에 대한 교육만족도 등은 향후 진행될 역학조사관 신규 교육프로그램의 개발에 도움이 될 것으로 판단되며, 국가 방역체계의 근간을 이룰 역학조사관의 역량강화에 크게 기여할 것으로 사료된다.

참고문헌

1. Korea Centers for Disease Control and Prevention. Infectious disease surveillance yearbook, 2015. Cheongju: Korea Centers for Disease Control and Prevention; 2016, p. 19 (Korean).

Volume: 39, Article ID: e2017032s

https://doi.org/10.4178/epih.e2017032

2. National Law Information Center. Infectious Disease Control and Prevention Act: Article 60-2; 2016 Jan 7 [cited 2017 Jul 31]. Available from: URL:http: //www.law.go.kr/main.html (Korean).
3. Oh SH. Men political science. Seoul: Parkyoungsa; 2010, p. 35-37 (Korean).
4. Kwon GY, Moon S, Kwak W, Gwack J, Chu C, Youn SK. Epidemic intelligence service officers and field epidemiology training program in Korea. Osong Public Health Res Perspect 2013;4:215-221.
5. Jun B. Middle East respiratory syndrome outbreak and infectious disease control in Korea. J Korean Med Assoc 2015;58:590-593 (Korean).
6. Korea Centers for Disease Control and Prevention. Infectious diseases control program guidelines. Cheongju: Korea Centers for Disease Control and Prevention; 2016, p. 95 (Korean).
7. Park NR, Jeong IS, Lee JG, Kim YT, Chun JH, Kim KS, et al. Evaluation of field epidemiology specialist training program based on the satisfaction and the changes of educational needs. Korean J Prev Med 2004;37: 80-87 (Korean).
8. Dongkuk University. Development of educational course and training manual for field epidemiologist training program during 2011-2015. Cheongju: Korea Centers for Disease Control and Prevention; 2011, p. 5 (Korean).
